# Facial contour modulation and skin tightening using 40.68-MHz unipolar radiofrequency

**DOI:** 10.1007/s10103-026-04956-8

**Published:** 2026-07-24

**Authors:** Jungbin Kim, Kisu Sung, Yerin Park, Kyu-Ho Yi

**Affiliations:** 1 Cheongdam JeunEx Clinic, Seoul, Republic of Korea; 2 Medical Research, Seoul, Republic of Korea; 3 You and I Clinic, Seoul, Republic of Korea

**Keywords:** Unipolar radiofrequency, 40.68 MHz, Facial tightening, Skin laxity, Elasticity, Three-dimensional volumetry

## Abstract

To evaluate changes in facial contour and skin elasticity following full-face treatment with 40.68-MHz unipolar radiofrequency (RF), and to assess its safety and tolerability in adults with mild-to-moderate facial laxity. This prospective single-arm interventional clinical study evaluated adults with mild-to-moderate facial laxity who underwent three full-face 40.68-MHz unipolar RF treatment sessions at 4-week intervals. The primary endpoint was the change in VECTRA-derived three-dimensional facial volumetric parameters from baseline to Week 20. Secondary outcomes included Cutometer-derived skin elasticity parameters (R2 and R5), Global Aesthetic Improvement Scale (GAIS), FACE-Q satisfaction scores, visual analog scale (VAS) pain scores, and adverse event monitoring. Of the 30 enrolled participants, 26 completed follow-up and were included in the final analysis. By Week 20, VECTRA 3D analysis demonstrated modest volume reductions in the cheek (− 2.2 mL), jowl (− 1.7 mL), and submental region (− 2.9 mL). Skin elasticity parameters increased over time, with R2 increasing from 0.62 to 0.69 and R5 from 0.53 to 0.59. GAIS improvement was observed in 88.5% of participants, and the mean FACE-Q score increased to 72.4 ± 10.1. Treatment was well tolerated, with low mean pain scores (VAS 1.2 ± 0.9). Only transient erythema and mild edema were reported, with no serious adverse events observed. Three sessions of 40.68-MHz unipolar RF were associated with modest measurable improvements in facial contour and skin elasticity that were maintained during the 20-week observation period, with good tolerability and no serious adverse events. Because this was an uncontrolled single-arm study, these findings should be interpreted as preliminary and require confirmation in controlled studies with longer follow-up.

## Introduction

Facial “sagging” or skin laxity is a visible consequence of progressive age-related change across multiple anatomic layers. With intrinsic aging and cumulative extrinsic exposures, the dermis demonstrates reduced structural integrity and elastic recoil, while deeper soft-tissue compartments contribute to contour change through gradual attenuation of supportive fibrous architecture and altered tissue mechanics. Clinically, these processes manifest as descent and loss of definition in the midface and jawline, formation of jowls, and worsening submental laxity—changes that are often perceived early and drive a growing demand for effective, low-downtime rejuvenation strategies. Reviews of facial RF technologies emphasize that laxity is not a single-layer problem and that successful nonsurgical tightening approaches must address dermal and subdermal tissue behavior rather than only superficial texture [1–3]. 

Radiofrequency (RF) has become a widely used non-ablative modality for facial rejuvenation because it can deliver controlled thermal energy to skin and subcutaneous soft tissue without epidermal ablation. The therapeutic premise is that heating in appropriate temperature ranges induces immediate, thermally mediated collagen structural alteration/contraction and triggers a wound-healing cascade that supports delayed remodeling, including neocollagenesis and reorganization of dermal architecture. Early clinical work demonstrated that non-ablative RF could improve laxity in aesthetically sensitive regions such as the periorbital area, and subsequent studies expanded clinical use to the face and neck [4–6]. Importantly, mechanistic support for remodeling has been provided by histologic and ultrastructural studies; in a seminal pilot study, RF-induced dermal changes consistent with collagen alteration and remodeling biology were documented following monopolar RF treatment [7]. More recent clinical and histometric investigations have continued to support measurable structural change after RF, consistent with progressive improvement over follow-up [8, 9]. 

Despite broad adoption, “RF” is not a single uniform technology. Clinical behavior depends on parameters such as frequency, applicator geometry, delivered power, coupling, and—critically—electrode configuration (e.g., monopolar/unipolar-type versus bipolar), which together determine heating distribution, depth profile, comfort, and safety. Technical and clinical reviews have highlighted that monopolar-type approaches can produce broader volumetric heating patterns but may require careful technique for tolerability, whereas bipolar systems tend to confine current paths more locally, often improving controllability and comfort at the expense of penetration depth depending on electrode spacing and design [2, 10, 11]. Accordingly, the evidence base for RF tightening is heterogeneous, spanning different device architectures and treatment protocols. Systematic reviews conclude that RF can improve facial and neck laxity with an overall acceptable safety profile, but also stress variability in outcomes reporting and the need for well-designed studies with standardized parameters and objective measurements [12, 13]. 

The importance of rigorous evaluation is particularly relevant in Asian populations and in patients with Fitzpatrick skin types III–IV, where clinicians often prioritize favorable tolerability and low risk of pigmentary sequelae while still seeking meaningful contour change. Clinical studies have reported the feasibility and efficacy of RF tightening in Asian skin, including monopolar RF for facial laxity and lower face rejuvenation, supporting RF as a practical option in this demographic [14–17]. Nevertheless, treatment goals in real-world practice often center on difficult-to-treat regions such as the jowl and submental area, and there remains ongoing interest in RF platforms and protocols that can deliver consistent tightening/lifting with minimal discomfort and minimal downtime [2, 18, 19]. 

A 40.68-MHz unipolar RF platform represents a distinct approach within aesthetic RF, with a treatment paradigm intended to achieve efficient thermal dosing over a short session while maintaining patient comfort. A clinical report describing a 40.68-MHz platform provides mechanistic framing for unipolar delivery and supports its use for rhytides and lax skin, but contemporary clinical data remain limited, particularly for standardized full-face protocols evaluated with objective quantitative endpoints [11, 20]. Therefore, the present prospective single-arm interventional clinical study was designed to evaluate the clinical safety, tolerability, and preliminary efficacy of a standardized, three-session full-face 40.68-MHz unipolar RF protocol for mild-to-moderate facial skin laxity in Fitzpatrick III–IV adults. A multimodal assessment strategy was used, including quantitative 3D stereophotogrammetry, Cutometer-derived elasticity parameters, aesthetic ratings, patient-reported FACE-Q outcomes, and systematic tolerability and safety monitoring.

Recent nonsurgical facial rejuvenation studies using collagen-stimulating injectables have similarly emphasized the value of 3D imaging, regional volumetric assessment, longitudinal follow-up, and patient-reported outcomes as complementary endpoints.[21, 22].

## Materials and methods

### Study design and participants

This was a prospective single-arm interventional clinical study evaluating a standardized full-face 40.68-MHz unipolar radiofrequency (RF, Ultratherma, Baren, Korea) treatment protocol in adults with mild-to-moderate facial skin laxity. Participants were enrolled prospectively and underwent three treatment sessions followed by scheduled outcome assessments through Week 20.

Eligible participants were adults aged 30–60 years with mild-to-moderate facial skin laxity who were willing to complete all treatment sessions and scheduled follow-up visits. Participants were excluded if they had received facial energy-based treatments within the previous 6 months or injectable procedures within the previous 12 months, had active dermatologic disease or infection in the treatment area, were pregnant, or had implanted electronic devices.

Participants were instructed to avoid additional facial energy-based procedures, injectable aesthetic treatments, or major changes in topical cosmetic regimens during the study period. Potential confounding factors such as concomitant aesthetic procedures, major weight changes, and relevant dermatologic events were reviewed during follow-up visits when available.

The study was conducted in accordance with the principles of the Declaration of Helsinki and was approved by the Public Institutional Review Board of the Republic of Korea (IRB No. P01-202509-01-018). Written informed consent was obtained from all participants before study participation. The protocol was not registered in a public clinical trial registry; this is acknowledged as a limitation of the study. Reporting was guided by relevant STROBE principles for transparent reporting of nonrandomized clinical research.

#### Endpoints

The primary endpoint was the change in VECTRA-derived 3D facial volumetric measurements from baseline to Week 20 in the predefined cheek, jowl, and submental regions. Secondary endpoints included changes in Cutometer-derived elasticity parameters (R2 and R5), GAIS improvement ratings, FACE-Q satisfaction scores, procedural pain assessed using VAS, downtime, and treatment-emergent adverse events.

### Treatment procedure

All treatments were performed using a radiofrequency device (UltraTherma; Jeunex Co., Ltd., Seoul, Republic of Korea). The 40.68-MHz lifting handpiece was used in unipolar mode. In this configuration, treatment was delivered through a single active applicator without the use of a separate patient grounding pad, which differs procedurally from conventional monopolar RF systems that deliver current between an active electrode and a return electrode.

A conductive high-frequency coupling cream was applied before each treatment session to facilitate consistent contact and glide between the applicator and the skin surface. The coupling cream was used as part of the standardized treatment protocol; its independent contribution to clinical outcomes was not evaluated in this study. Treatments were performed three times at 4-week intervals (Week 0, Week 4, and Week 8). The treated regions included the midface, perioral region, jawline, and submental area.

Treatment parameters were standardized across participants. RF energy was delivered at a frequency of 40.68 MHz using the lifting handpiece in unipolar mode. The device output was set according to manufacturer-defined treatment levels: Level 6 for the midface, perioral region, and jawline, and Level 8 for the submental area to account for regional differences in tissue thickness. These levels represent device-defined intensity settings rather than direct real-time measurements of energy delivered to tissue; therefore, the exact amount of energy delivered to the tissue could not be quantified in this study.

The handpiece was applied with a slow, continuous sliding motion rather than a stamping technique. Three to five overlapping passes were performed over each treatment region, and the total treatment time was approximately 10 min per session. No topical anesthesia, epidermal cooling, or systemic analgesics were used. The clinical endpoint was uniform warmth with mild transient erythema. Formal intradermal thermometry was not performed, and surface temperature monitoring was not available for quantitative analysis; this limitation is acknowledged in the Discussion. The standardized treatment protocol and device settings by anatomical region are summarized in Table 1.

**Table 1:** Treatment protocol and device settings by anatomical region

Region	RF frequency	Mode	Device-defined level	Handpiece movement	Passes	Approximate session time	Anesthesia/cooling	Clinical endpoint
Midface	40.68 MHz	Unipolar	Level 6	Slow continuous sliding motion	3–5 overlapping passes	Included within approximately 10 min total full-face treatment time	None	Uniform warmth with mild transient erythema
Perioral region	40.68 MHz	Unipolar	Level 6	Slow continuous sliding motion	3–5 overlapping passes	Included within approximately 10 min total full-face treatment time	None	Uniform warmth with mild transient erythema
Jawline	40.68 MHz	Unipolar	Level 6	Slow continuous sliding motion	3–5 overlapping passes	Included within approximately 10 min total full-face treatment time	None	Uniform warmth with mild transient erythema
Submental region	40.68 MHz	Unipolar	Level 8	Slow continuous sliding motion	3–5 overlapping passes	Included within approximately 10 min total full-face treatment time	None	Uniform warmth with mild transient erythema

Device-defined levels indicate manufacturer-defined intensity settings and do not represent direct real-time measurements of energy delivered to tissue

Assessments were conducted at baseline (Week 0; prior to the first treatment), Week 12 (4 weeks after the final treatment), and Week 20 (12 weeks after the final treatment)

#### Outcome measures

1. Three-Dimensional (3D) Imaging Analysis

Three-dimensional facial imaging was performed using the VECTRA system (Canfield Scientific, Inc., Parsippany, NJ, USA). Images were obtained at baseline, Week 12, and Week 20 under standardized acquisition conditions, with participants positioned in a reproducible upright posture and instructed to maintain a neutral facial expression. Quantitative volumetric changes were calculated relative to baseline for predefined regions of interest, including the bilateral midface (cheek), jowl region, and submental region, and were expressed as changes in volume (mL).

Image alignment and volumetric comparison were performed using baseline images as the reference. The VECTRA-derived measurements were interpreted as changes in surface topography and regional contour rather than direct evidence of fat reduction or tissue remodeling. Formal repeated-measurement reproducibility testing and a predefined minimal clinically important difference for these regional volumetric changes were not established in the present study; therefore, small volumetric changes were interpreted cautiously.

2) Skin Elasticity Assessment

Skin elasticity was measured using a Cutometer device. The parameters analyzed were R2 (gross elasticity) and R5 (net elasticity). Measurements were performed at the scheduled study visits using the same general assessment protocol across time points. Because intra-session reliability testing and formal test-retest variability analysis were not performed in this study, the clinical interpretation of small changes in Cutometer-derived parameters was made cautiously.

3) Aesthetic Scale and Patient-Reported Outcomes

Two independent clinicians who did not participate in the treatment procedures evaluated standardized clinical photographs using a 5-point Global Aesthetic Improvement Scale (GAIS). Evaluators were blinded to participant identifiers and treatment details. Because GAIS assessment requires comparison of baseline and follow-up appearance, complete temporal blinding was not feasible. Formal inter-rater reliability statistics were not available for this analysis and are acknowledged as a limitation.

Participants also completed the FACE-Q questionnaire assessing satisfaction with facial appearance. Scores were converted to Rasch-transformed scores (0–100), with higher scores indicating greater satisfaction. Because the study did not include a sham or comparator group, patient-reported outcomes were interpreted as supportive exploratory outcomes rather than independent proof of treatment efficacy.

4) Tolerability and Safety

Procedural discomfort was assessed immediately after each treatment session using a 0–10 visual analog scale (VAS). Adverse events were recorded at each study visit, including erythema, edema, burns, pigmentary changes, nodules, and sensory changes. Downtime was defined as the number of days required for participants to return to normal daily activities.

### Statistical analysis

Statistical analyses were performed using SPSS software (version 27.0; IBM Corp., Armonk, NY, USA). Continuous variables were summarized as mean ± standard deviation (SD), and categorical variables were presented as frequencies and percentages. Changes over time were analyzed using repeated-measures analysis of variance (ANOVA). If the assumption of normality was not met, the Friedman test was applied, followed by Wilcoxon signed-rank tests for pairwise comparisons. All statistical tests were two-tailed, and a P value of < 0.05 was considered statistically significant.

The primary analysis included participants who completed the scheduled follow-up assessments. Missing follow-up data from participants who did not complete the study were not imputed. No formal sample size calculation was performed; therefore, the study should be interpreted as exploratory. Because the available dataset was analyzed using summarized repeated-measures comparisons, effect sizes and 95% confidence intervals were not available for all endpoints; this limitation is acknowledged when interpreting the magnitude and precision of the observed changes.

## Results

### Participant Flow and Baseline Characteristics

Thirty participants were enrolled, and 26 completed all treatment sessions and follow-up visits and were included in the final analysis. The final analyzed cohort included 21 females and 5 males, with a mean age of 45.1 ± 7.3 years (range, 32–59 years), and all participants had Fitzpatrick skin types III–IV. Four participants did not complete follow-up and were therefore excluded from the final analysis. No serious adverse events were recorded among the analyzed participants. Because detailed outcome data were not available for participants who did not complete follow-up, missing data were not imputed.

### Three-dimensional (3D) imaging analysis

Three-dimensional VECTRA analysis showed measurable volumetric reductions in the bilateral midface (cheek), jowl, and submental regions by Week 12, with modest additional change at Week 20 (Table 2). The largest absolute reduction at Week 20 was observed in the submental region. These VECTRA-derived changes were interpreted as quantitative changes in surface contour and regional topography rather than direct evidence of tissue composition change or clinically meaningful lifting.

**Table 2:** VECTRA volumetric outcomes over time (*n* = 26)

Region	Baseline	Week 12	Week 20	Δ (W0 ◊ W 20)	*P* value*
Cheek	24.6 ± 5.4	23.0 ± 5.2	22.4 ± 5.0	−2.2	< 0.01
Jowl	10.5 ± 3.8	9.2 ± 3.6	8.8 ± 3.5	−1.7	< 0.01
Submental	17.8 ± 6.0	15.6 ± 5.8	14.9 ± 5.6	−2.9	< 0.001

Data are presented as mean ± standard deviation (SD). Δ indicates the change from baseline (Week 0) to Week 20. W0, Week 0; W12, Week 12; W20, Week 20

### Skin elasticity

Cutometer measurements showed increases in skin elasticity parameters over time. R2 (gross elasticity) increased from 0.62 ± 0.05 at baseline to 0.67 ± 0.05 at Week 12 and 0.69 ± 0.06 at Week 20 (*P* < 0.01). R5 (net elasticity) increased from 0.53 ± 0.06 at baseline to 0.57 ± 0.06 at Week 12 and 0.59 ± 0.07 at Week 20 (*P* < 0.01). Because intra-session reliability testing was not performed, the clinical significance of these changes should be interpreted cautiously.

### Aesthetic improvement

#### Gais

**Fig. 1:**
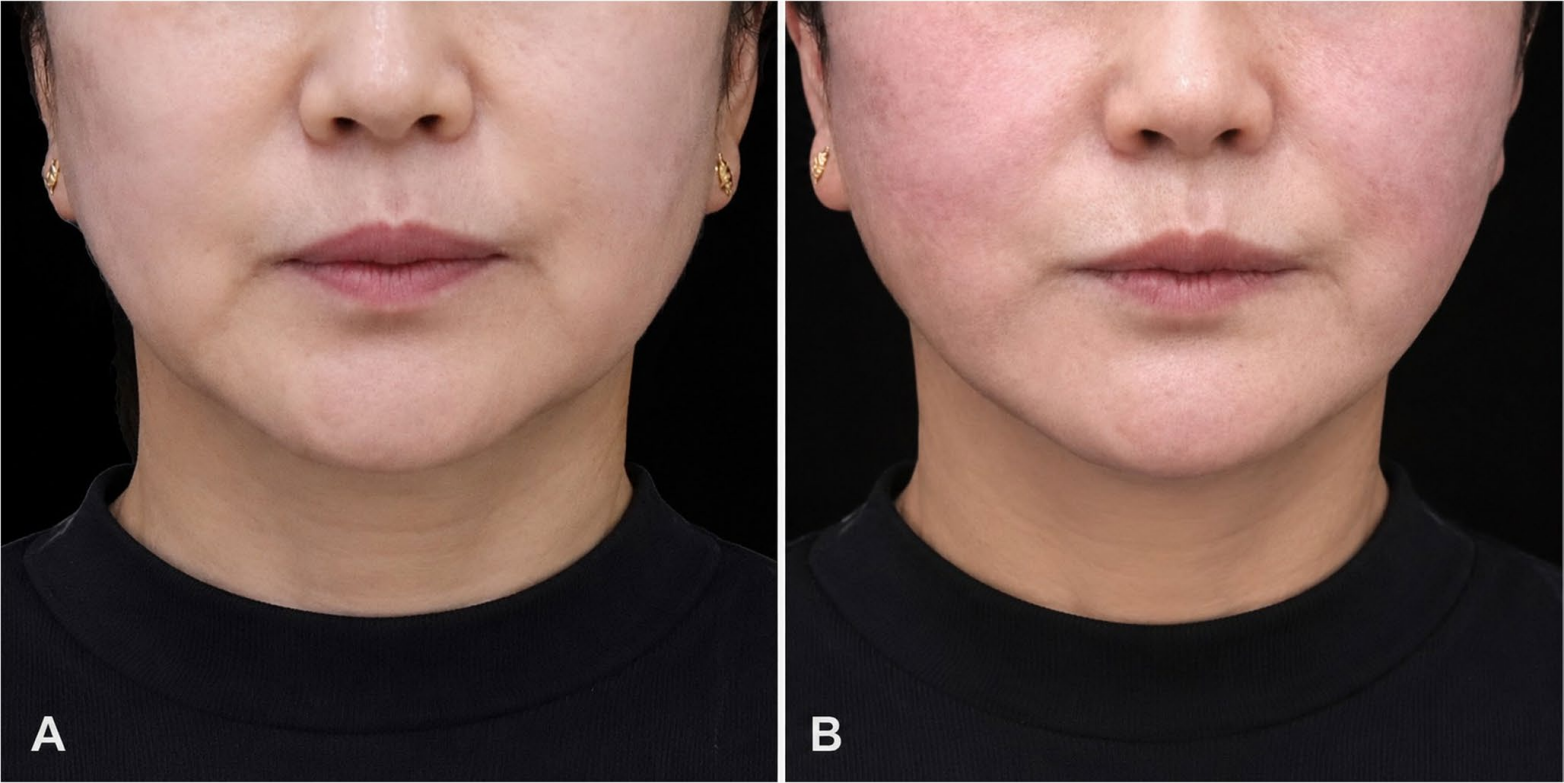
Representative clinical photographs (42-year-old participant). (**A**) Baseline (Week 0) and (**B**) Week 20 after three full-face 40.68-MHz unipolar RF sessions. The Week 20 image demonstrates subtle contour refinement, with mild apparent improvement in midface support and jawline/submental definition. These photographs are presented as representative clinical examples and should be interpreted together with quantitative outcome measures

**Fig. 2:**
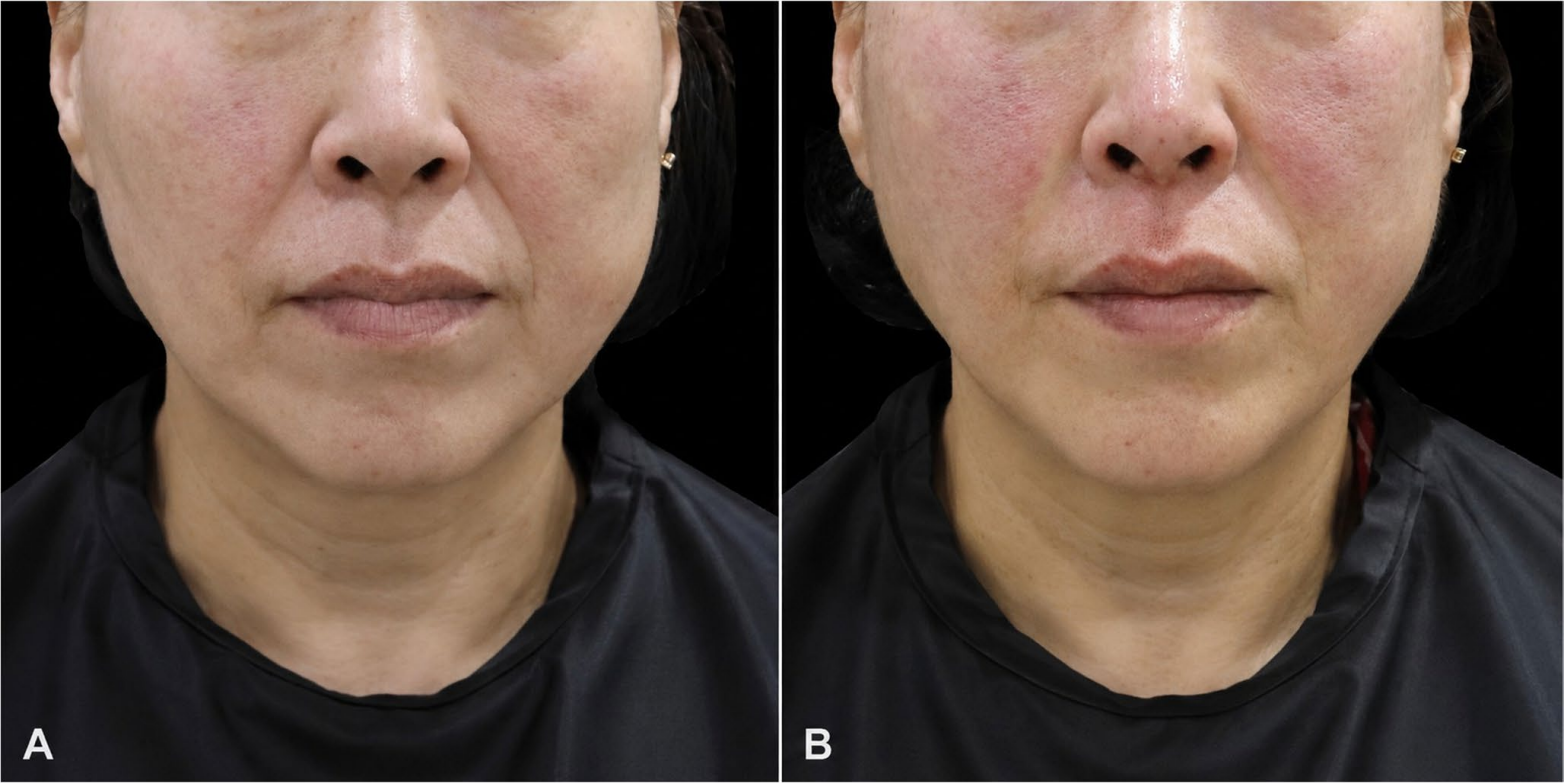
Representative clinical photographs (50-year-old participant). (**A**) Baseline (Week 0) and (**B**) Week 20 after three full-face 40.68-MHz unipolar RF sessions. The Week 20 image shows modest apparent contour refinement, including mild improvement in the jawline and submental outline. The clinical photographs are intended to illustrate representative changes and do not independently establish clinical efficacy

At Week 12, improvement (≥ “Improved”) was observed in 80.8% of participants. At Week 20, the improvement rate increased to 88.5%, with a greater proportion of participants rated as “Much improved” or “Very much improved.” These ratings were performed by two independent clinicians who were not involved in treatment; however, because paired photographic assessment requires comparison with baseline appearance, the GAIS results should be interpreted as supportive and exploratory. Figures 1 and 2 present representative Week 20 cases rated as “Improved”.

#### FACE-Q

Mean FACE-Q satisfaction scores increased from 55.2 ± 9.4 at baseline to 66.5 ± 9.6 at Week 12 and 72.4 ± 10.1 at Week 20 (*P* < 0.001). These patient-reported outcomes suggest improved satisfaction during follow-up but should be interpreted cautiously because the study did not include a sham or comparator group.

#### Tolerability and safety

The mean procedural pain score was 1.2 ± 0.9 on the visual analog scale. Transient erythema was observed in 18 participants (69.2%) and resolved within several hours. Mild edema occurred in 3 participants (11.5%) and resolved within 24 h. No burns, post-inflammatory hyperpigmentation, nodules, sensory changes, or fat atrophy were observed. No downtime was reported. All participants returned to normal daily activities immediately after each treatment session.

## Discussion

In this prospective single-arm interventional study, three sessions of full-face 40.68-MHz unipolar radiofrequency (RF) were associated with modest measurable improvements in facial surface contour and skin elasticity in adults with mild-to-moderate facial laxity. Objective 3D stereophotogrammetry showed regional volumetric reductions in the cheek, jowl, and submental regions, while Cutometer parameters, GAIS ratings, and FACE-Q satisfaction scores improved during the 20-week observation period. Because the study lacked a sham or active-comparator group, these findings should be interpreted as preliminary associations rather than definitive evidence of treatment efficacy.

The observed time course may be consistent with mechanisms previously reported for nonablative RF tightening, including early thermally mediated collagen structural changes and delayed extracellular matrix remodeling. Histologic and ultrastructural studies of monopolar RF have reported collagen fibril alteration, inflammatory signaling, and increased type I collagen mRNA expression after treatment [7]. However, the present study did not include histologic, biochemical, molecular, or intradermal thermal measurements. Therefore, collagen remodeling and neocollagenesis should be regarded as plausible mechanisms based on prior literature rather than mechanisms directly demonstrated by the present data.

The VECTRA-derived volume reductions should be interpreted as changes in surface topography and regional contour rather than direct proof of adipose reduction, tissue lifting, or structural remodeling. Although RF can heat subcutaneous tissue under certain parameters, the present study did not include ultrasound measurement of subcutaneous fat thickness, histologic analysis, or direct thermal mapping. In addition, minimal clinically important differences for VECTRA-derived facial volumetric changes have not been firmly established for this indication. Therefore, the clinical relevance of small volumetric changes should be interpreted cautiously and in conjunction with other objective and subjective outcomes.

The clinical behavior of RF depends not only on frequency but also on electrode configuration, applicator geometry, delivered power, coupling, cooling, and treatment technique. Conventional monopolar RF systems deliver energy between an active treatment electrode and a return electrode or grounding pad, producing a broader current pathway through tissue. Bipolar RF systems deliver current between two electrodes within the treatment handpiece, with heating patterns influenced by electrode spacing and geometry. In contrast, the 40.68-MHz unipolar configuration used in the present study was applied through a single active applicator without a separate grounding pad, and the handpiece was moved in a slow continuous sliding motion rather than applied as a stamping technique.

**Fig. 3:**
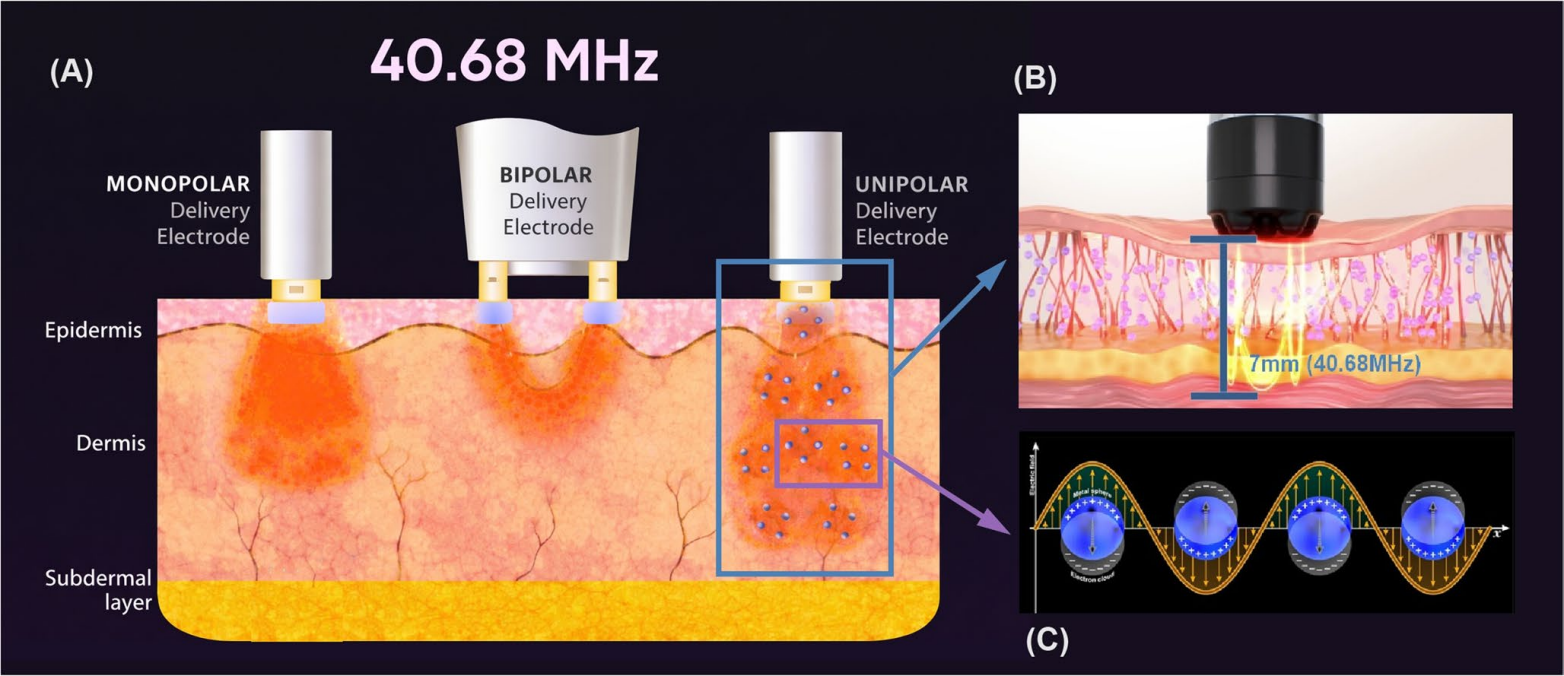
Schematic comparison of RF electrode configurations and conceptual energy distribution patterns. (**A**) Conceptual illustration of monopolar, bipolar, and 40.68-MHz unipolar RF delivery across the epidermal, dermal, and subdermal layers; shading denotes representative regions of energy deposition/heating. (**B**) Schematic of unipolar application with continuous sliding movement of the handpiece over the skin surface. (**C**) Conceptual illustration of polarization/dipole response in an oscillating electromagnetic field during high-frequency RF exposure. This figure is schematic and is not intended to represent measured tissue heating depth in the present study

Recent RF platforms illustrate the diversity of contemporary device designs. For example, dual-frequency monopolar RF platforms such as XERF have been described as combining 6.78 MHz and 2 MHz frequencies with impedance feedback and cooling strategies, representing a different technical approach from the fixed 40.68-MHz unipolar protocol evaluated in the present study (Fig. 3). These comparisons are useful for contextualization, but direct efficacy or safety comparisons cannot be made because treatment frequency, electrode design, thermal monitoring, cooling, and clinical endpoints differ substantially across platforms [23, 24].

These technical distinctions may partly explain differences in tolerability and treatment experience across RF platforms. In the present cohort, treatment was performed without topical anesthesia, cooling, or systemic analgesics, and the mean pain score was low. However, because this study did not directly compare different RF systems or quantify real-time tissue energy delivery, no conclusion can be drawn regarding superiority over other RF technologies.

A conductive coupling cream was used consistently as part of the treatment protocol to facilitate applicator contact and glide. However, the present study was not designed to evaluate the independent contribution of the coupling cream to clinical outcomes. Therefore, no conclusion can be drawn regarding any separate therapeutic effect of the cream, and the observed outcomes should be attributed only to the overall treatment protocol rather than to the coupling medium itself.

The safety findings were favorable within the 20-week observation period. Only transient erythema and mild edema were reported, and no burns, pigmentary change, nodules, sensory changes, clinically evident fat atrophy, or downtime were observed among participants who completed follow-up. These findings are consistent with prior reports suggesting that RF-based tightening procedures can have an acceptable safety profile when appropriately applied [13]. However, the modest sample size limits detection of uncommon adverse events, and longer follow-up is needed to better characterize delayed safety outcomes.

The Fitzpatrick III–IV cohort is also clinically relevant because pigmentary safety and tolerability are important considerations in Asian skin. RF is generally considered less chromophore-dependent than light-based devices, which may be advantageous in darker phototypes; however, skin type alone does not determine response. Differences in dermal thickness, baseline laxity, pigmentation tendency, and patient expectations may influence perceived and measured outcomes. Because the present study included only Fitzpatrick III–IV participants and did not include a non-Asian or lighter-phototype comparator group, no conclusion can be drawn regarding differential efficacy or safety between Asian and non-Asian skin.

Several limitations should be acknowledged. First, this was a single-arm, nonrandomized study without a sham or active-comparator group; therefore, causal inference is limited, and placebo effects or expectation bias may have influenced subjective outcomes such as GAIS and FACE-Q. Second, the sample size was modest, and no formal sample size calculation was performed, so the study should be interpreted as exploratory. Third, four enrolled participants did not complete follow-up, and missing data were not imputed. Fourth, treatment intensity was recorded using manufacturer-defined device levels rather than direct real-time measurements of energy delivered to tissue. Formal intradermal thermometry and quantitative surface temperature monitoring were not performed, limiting interpretation of dose-response and thermal mechanisms. Fifth, the VECTRA-derived volumetric changes reflect surface topography and may be influenced by positioning, registration, and measurement variability; a predefined minimal clinically important difference was not established. Sixth, intra-session Cutometer reliability and formal inter-rater reliability statistics for GAIS were not available. Seventh, the study did not include ultrasound, histology, biochemical analysis, or molecular testing; therefore, collagen remodeling, neocollagenesis, and tissue tightening mechanisms remain inferential rather than directly demonstrated. Finally, follow-up was limited to Week 20, which does not allow conclusions regarding long-term durability. Future randomized or sham-controlled studies should include larger sample sizes, objective thermal monitoring, standardized reproducibility testing, longer follow-up, and comparator arms to better define clinical efficacy, safety, and dose-response relationships.

## Conclusion

In this prospective single-arm interventional study, three sessions of full-face 40.68-MHz unipolar radiofrequency were associated with modest measurable improvements in facial surface contour and skin elasticity in adults with mild-to-moderate facial laxity, with good tolerability and no serious adverse events observed during the 20-week follow-up period. These findings provide preliminary clinical evidence for this underreported RF frequency and treatment configuration using multimodal assessment, including 3D volumetric analysis, elasticity measurements, aesthetic ratings, patient-reported outcomes, and safety monitoring. However, because the study lacked a sham or comparator group and did not include direct thermal, histologic, or molecular confirmation, the findings should be interpreted cautiously. Larger controlled studies with longer follow-up are required to confirm efficacy, determine clinical significance, and clarify mechanisms of action. 

## Data Availability

The data that support the findings of this study are available on request from the corresponding author. The data are not publicly available due to privacy or ethical restrictions.

## References

[1] Atiyeh BS, Dibo SA (2009) Nonsurgical nonablative treatment of aging skin: radiofrequency technologies between aggressive marketing and evidence-based efficacy. Aesthetic Plast Surg May 33(3):283–294. 10.1007/s00266-009-9361-9 10.1007/s00266-009-9361-9 19437070

[2] Gentile RD, Kinney BM, Sadick NS (May 2018) Radiofrequency Technology in Face and Neck Rejuvenation. *Facial Plast Surg Clin North Am*. 26(2):123–134. 10.1016/j.fsc.2017.12.003 10.1016/j.fsc.2017.12.003 29636146

[3] Lyu J-J, Liu S-X (2022) Radiofrequency in facial rejuvenation. Int J Dermatology Venereol 5(02):94–100

[4] Abraham MT, Chiang SK, Keller GS, Rawnsley JD, Blackwell KE, Elashoff DA (2004) Clinical evaluation of non-ablative radiofrequency facial rejuvenation. J Cosmet Laser Ther Nov 6(3):136–144. 10.1080/14764170410023802 10.1080/14764170410023802 15545097

[5] Abraham MT, Vic Ross E (2005) Current concepts in nonablative radiofrequency rejuvenation of the lower face and neck. Facial Plast Surg Feb 21(1):65–73. 10.1055/s-2005-871765 10.1055/s-2005-871765 15988658

[6] Fitzpatrick R, Geronemus R, Goldberg D, Kaminer M, Kilmer S, Ruiz-Esparza J (2003) Multicenter study of noninvasive radiofrequency for periorbital tissue tightening. Lasers Surg Med 33(4):232–242. 10.1002/lsm.10225 14571447 10.1002/lsm.10225

[7] Zelickson BD, Kist D, Bernstein E et al (2004) Histological and ultrastructural evaluation of the effects of a radiofrequency-based nonablative dermal remodeling device: a pilot study. Arch Dermatol Feb 140(2):204–209. 10.1001/archderm.140.2.204 10.1001/archderm.140.2.204 14967794

[8] El-Domyati M, El-Ammawi TS, Medhat W et al (2011) Radiofrequency facial rejuvenation: evidence-based effect. J Am Acad Dermatol 64(3):524–535 21315951 10.1016/j.jaad.2010.06.045 PMC6541915

[9] Suh DH, Ahn HJ, Seo JK, Lee SJ, Shin MK, Song KY (2020) Monopolar radiofrequency treatment for facial laxity: Histometric analysis. J Cosmet Dermatol Sep 19(9):2317–2324. 10.1111/jocd.13449 10.1111/jocd.13449 32319176

[10] Carruthers J, Fabi S, Weiss R (2014) Monopolar radiofrequency for skin tightening: our experience and a review of the literature. Dermatol Surg Dec 40(Suppl 12):S168–S173. 10.1097/dss.0000000000000232 10.1097/DSS.0000000000000232 25417570

[11] Weiss RA (2013) Noninvasive radio frequency for skin tightening and body contouring. Semin Cutan Med Surg 32(1):9–17 24049924

[12] Austin GK, Struble SL, Quatela VC (Jan 2022) Evaluating the effectiveness and safety of radiofrequency for face and neck rejuvenation: A systematic review. Lasers Surg Med 54(1):27–45. 10.1002/lsm.23506 10.1002/lsm.23506 34923652

[13] Rohrich RJ, Schultz KP, Chamata ES, Bellamy JL, Alleyne B (2022) Minimally invasive approach to skin tightening of the face and body: systematic review of monopolar and bipolar radiofrequency devices. Plast Reconstr Surg 150(4):771–780 35877937 10.1097/PRS.0000000000009535

[14] Kushikata N, Negishi K, Tezuka Y, Takeuchi K, Wakamatsu S (2005) Non-ablative skin tightening with radiofrequency in Asian skin. Lasers Surg Med Feb 36(2):92–97. 10.1002/lsm.20136 10.1002/lsm.20136 15704167

[15] Suh DH, Ahn HJ, Seo JK, Lee SJ (2020) Monopolar radiofrequency treatment for facial laxity. J Cosmet Dermatol 19:2317–2324 32319176 10.1111/jocd.13449

[16] Suh DH, Chang KY, Ryou JH, Lee SJ, Kim HS (2011) Monopolar radio-frequency treatment in Asian skin: a questionnaire-based study. J Cosmet Laser Ther Jun 13(3):126–129. 10.3109/14764172.2011.581288 10.3109/14764172.2011.581288 21609216

[17] Wanitphakdeedecha R, Yogya Y, Yan C et al (2022) Efficacy and Safety of Monopolar Radiofrequency for Treatment of Lower Facial Laxity in Asians. Dermatol Ther (Heidelb) Nov 12(11):2563–2573. 10.1007/s13555-022-00817-8 10.1007/s13555-022-00817-8 PMC9588109 36166188

[18] Chawvavanich P, Singthong S, Intarachaieua K (2022) Effectiveness and side effects of bipolar radiofrequency to treat submental laxity. J Cosmet Dermatol 21(10):4392–4397 35255190 10.1111/jocd.14898

[19] Park J-H, Kim J-I, Park HJ, Kim W-S (2016) Evaluation of safety and efficacy of noninvasive radiofrequency technology for submental rejuvenation. Lasers Med Sci 31(8):1599–1605 27402002 10.1007/s10103-016-2023-7

[20] Friedman DJ, Gilead LT (2007) The use of hybrid radiofrequency device for the treatment of rhytides and lax skin. Dermatol Surg 33(5):543–551 17451576 10.1111/j.1524-4725.2007.33112.x

[21] Ponzo M, Lombardi M, Avvedimento S, D’Alessio FM, Cavallini M, Santorelli A (2026) Efficacy and Safety of Poly-L-lactic Acid for Midface Rejuvenation: A 12-Month Split-face Controlled Evaluation With 3D Imaging and Patient-reported Outcomes. Aesthet Surg J 46(6):663–671. 10.1093/asj/sjag039 41662390 10.1093/asj/sjag039

[22] Ponzo M, La Padula S, Lombardi M, Cavallini M, Santorelli A (2025) Pilot Study: Efficacy and Safety of Polycaprolactone Collagen Stimulator for Middle Third of the Face. Plast Reconstr Surg Glob Open 13(11):e7285. 10.1097/GOX.0000000000007285 41210393 10.1097/GOX.0000000000007285 PMC12594308

[23] Adatto MA, Adatto-Neilson RM, Morren G (2014) Reduction in adipose tissue volume using a new high-power radiofrequency technology combined with infrared light and mechanical manipulation for body contouring. Lasers Med Sci Sep 29(5):1627–1631. 10.1007/s10103-014-1564-x 10.1007/s10103-014-1564-x PMC4149887 24687404

[24] Franco W, Kothare A, Ronan SJ, Grekin RC, McCalmont TH (2010) Hyperthermic injury to adipocyte cells by selective heating of subcutaneous fat with a novel radiofrequency device: feasibility studies. Lasers Surg Med Jul 42(5):361–370. 10.1002/lsm.20925 10.1002/lsm.20925 20583242

